# Serum amyloid A in children and adolescents: association with overweight and carotid intima-media thickness

**DOI:** 10.31744/einstein_journal/2023AO0251

**Published:** 2023-06-06

**Authors:** Maria Vitória Mareschi Barbosa, João Carlos Pina Faria, Stephanie Ramos Coelho, Fernando Luiz Affonso Fonseca, Andrea Paula Kafejian Haddad, Fabíola Isabel Suano de Souza, Roseli Oselka Saccardo Sarni

**Affiliations:** 1 Centro Universitário FMABC Santo André SP Brazil Centro Universitário FMABC, Santo André, SP, Brazil.; 2 Universidade Federal de São Paulo São Paulo SP Brazil Universidade Federal de São Paulo, São Paulo, SP, Brazil.

**Keywords:** Serum amyloid A protein, Carotid intima-media thickness, Apolipoproteins, Pediatric obesity, Overweight, Child

## Abstract

**Objective:**

To compare serum amyloid A concentrations between overweight and eutrophic children and adolescents and to relate it to lipid profiles, glucose tolerance, and carotid intima-media thickness.

**Methods:**

One hundred children and adolescents (mean age: 10.8±3.16 years) were included and divided into two groups: overweight and non-overweight. The following were evaluated: Z-score body mass index, carotid intima-media thickness, lipid metabolism biomarkers (lipid profile and apolipoproteins A1 and B), inflammatory biomarkers (ultra-sensitive C-reactive protein and serum amyloid A), and glucose homeostasis model assessment of insulin resistance.

**Results:**

The groups were homogeneous in age, sex, and pubertal stage. Higher levels of triglycerides, apolipoprotein B, homeostasis model assessment of insulin resistance, ultrasensitive C-reactive protein, serum amyloid A, and carotid intima-media thickness were observed in the overweight group. In the multivariate analysis, age (OR=1.73; 95%CI: 1.16-2.60, p=0.007), Z-score body mass index (OR=3.76; 95%CI: 1.64-8.59, p=0.002), apolipoprotein-B (OR=1.1; 95%CI: 1.01-1.2, p=0.030), and carotid intima-media thickness (OR=5.00; 95%CI: 1.38-18.04, p=0.014) were independently associated with serum amyloid A levels above the fourth quartile of the studied sample (>9.4mg/dL).

**Conclusion:**

Overweight children and adolescents had higher serum amyloid A concentrations than eutrophic children. There was an independent association between higher concentrations of serum amyloid A and Z-score, body mass index, apolipoprotein B, and carotid intima-media thickness, indicating the importance of this inflammatory biomarker in identifying the early risk of atherosclerosis.

## Highlights

■ Serum amyloid A is an acute phase inflammatory protein.

■ Excess weight causes meta-inflammation with a consequent increase in the synthesis of C-reactive protein and serum amyloid A.

■ Carotid intima-media thickness measurement evaluates atherosclerosis before atheromatous plaque formation.

**Figure:**
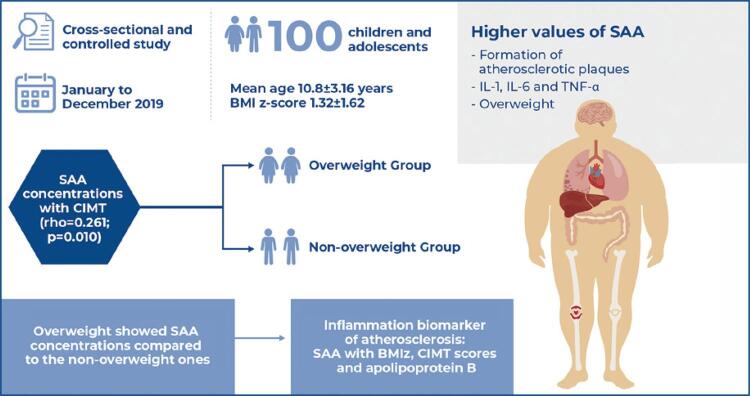

## INTRODUCTION

The worldwide prevalence of childhood obesity has increased ten-fold in the last 40 years.^(1)^Studies have revealed that approximately 30% of children aged five-nine years and 19.4% of adolescents in Brazil were overweight.^(2)^

Obesity is associated with chronic systemic inflammation, and serum inflammation markers, such as serum amyloid A (SAA) and ultra-sensitive C-reactive protein (us-CRP), are independent risk factors for cardiovascular disease (CVD).^(3)^

Serum amyloid A is a non-specific acute-phase protein with production related to inflammatory stimuli by cytokines, including IL-1, IL-6, and TNF-α. However, in contrast to us-CRP, which is primarily expressed in the human liver, SAA is expressed in both the liver and adipose tissues and is thus considered an adipokine.^(4)^

The increased expression of SAA by adipocytes in patients with obesity suggests that SAA might play a critical role in local and systemic inflammation, in the production of free fatty acids, and may represent a direct link between obesity and its comorbidities, such as insulin resistance and atherosclerosis.^(5)^

Additionally, a meta-analysis and systematic review has demonstrated that SAA concentrations in adults are positively associated with BMI and that weight loss is associated with a reduction in its levels.^(6)^

## OBJECTIVE

To compare the serum amyloid A concentrations in overweight children and adolescents with those in healthy individuals and relate it to lipid profile, glucose tolerance, and carotid intima-media thickness.

## METHODS

Through a cross-sectional and controlled study conducted between January and December 2019, 50 overweight and obese children and adolescents were included in the study and followed up at the outpatient clinic of the *Centro Universitário* FMABC in Santo André, (SP), Brazil. Fifty healthy and eutrophic volunteer children and adolescents, matched by sex and age, from a teaching institution located in the same municipality, were also included. Written consent was obtained from all participants. The children were accompanied by their parents.

Exclusion criteria included patients with chronic diseases (except asthma and rhinitis) or acute infections, and those using hormonal and non-hormonal anti-inflammatory drugs or immunosuppressants in the three months preceding the collection of biochemical tests.

Weight and waist height measurements were obtained at our institution. Z-score body mass index (BMIz) was calculated using the cutoff points recommended by the World Health Organization (WHO). Children with a BMIz between -2 and +1, between +1 and +2, between +2 and +3, and above 3 were classified as eutrophic, overweight, obese, and severely obese, respectively. The waist-to-height ratio (WHtR) was classified as altered when the value was equal to or greater than 0.5.

Fifteen mL of venous blood was collected by nurses from the laboratory of the *Centro Universitário* FMABC, in the morning, after a 12-hour fast. Lipid profile was measured by colorimetric enzymatic method with spectrophotometric reading (Cobas Integra 400 plus kit and Roche/Hitachi Modular kit). Concentrations of apolipoproteins A1 and B, insulin, blood glucose, us-CRP, and serum amyloid A were measured using Elisa (kit for Human apo A1 and B, Mabtech, Cincinnati, USA), chemiluminescence (Roche/Elecsys kit, 2010), colorimetric enzymatic method with spectrophotometric reading (Roche/Hitachi Modular kit), immunoturbidimetry (Roche/Hitachi 902 kit), and Elisa (FineTest kit), respectively.

Values of non-HDL-C (NHDL-C) and the ratios of total cholesterol/HDL-C, LDL-C/HDL-C, Apolipoprotein B/Apolipoprotein A1, and Triglycerides/HDL_,_ LDL-c/Apolipoprotein B were calculated. For the classification of the lipid profile, the cutoff points proposed by the American Heart Association were adopted*.*^(7)^ The following were considered altered: total cholesterol ≥170mg/dL, LDL-C ≥110mg/dL, HDL-C <40mg/dL, triglycerides ≥90mg/dL, apolipoprotein A1 <120mg/dL, and apolipoprotein B ≥90mg/dL. The Homeostasis Model Assessment of Insulin Resistance (HOMA-IR) value was calculated using fasting glucose and insulin values. HOMA-IR and serum amyloid A concentrations were analyzed as continuous variables.

Carotid intima-media thickness (CIMT) in the common carotid arteries was measured using B-mode Doppler ultrasonography performed by a Vascular Surgery Specialist. A Toshiba device (Model UJUR-590A) was used for measurements. The final CIMT measurement was obtained as the mean of five measurements taken in the right and left common carotid arteries. The measurements were performed as proposed by the Association for European Pediatric Cardiology^(8)^and the CIMT was analyzed as a continuous variable.

Data were entered and consolidated in an Excel spreadsheet (Office^®^), and analyses were performed using the SPSS 25.0 statistical package. Qualitative variables were compared using the χ^2^ test. The parametric distributions were presented as mean±standard deviation and compared using the Student *t*-test, while those with non-parametric distribution were presented as the median and compared using the Mann-Whitney test. For the correlation analysis, Pearson and Spearman’s tests were used for continuous variables with parametric and non-parametric distributions, respectively. The univariate ROC curve was used for the discriminatory analysis of biochemical variables with respect to nutritional status, and the multivariate ROC curve was used for variables associated with serum amyloid A concentrations (>fourth quartile, >9.4mg/dL) and CIMT (mean carotids D and E) (>fourth quartile, >0.3mm).

This study was approved by the Research Ethics Committee of the *Centro Universitário* FMABC, CAAE: 91719518.6.0000.0082; # 2.853.273.

## RESULTS

The mean age and BMI z-score of the assessed individuals (n=100) were 10.82±3.16 years and 1.32±1.62, respectively. There was no difference in sex, age, pubertal stage, or lipid profile classification between the overweight and non-overweight.

Table 1 shows the general characteristics and lipid profiles of the study population, stratified according to nutritional status.

**Table 1:** General characteristics and lipid profile of the population studied, stratified according to nutritional status

Variables		Overweight	Non-overweight	p value*
Sex	Male	25 (50)	25 (50)	0.579
Age	<10 years	21 (42.0)	21 (42.0)	0.580
Waist-to-height ratio	>0.5	40 (80.0)	5 (10.0)	<0.001
Pubertal development	Prepubescent	22 (44.0)	24 (48.0)	0.841
Total cholesterol	>170mg/dL	11 (22.0)	14 (28.0)	0.645
LDL-C	>110mg/dL	9 (18.0)	9 (18.0)	0.602
HDL-C	<45mg/dL	28 (56.0)	21 (42.0)	0.230
Non-HDL-C	>120mg/dL	13 (26.0)	13 (26.0)	0.590
Triglycerides	>90mg/dL	22 (44.0)	13 (26.0)	0.093
Apolipoprotein-B	>90mg/dL	21 (42.0)	9 (18.0)	0.016
Apolipoprotein-A1	<120mg/dL	18 (36.0)	10 (20.0)	0.118

* Significance level of the χ^2^ test.

**Figure 1:**
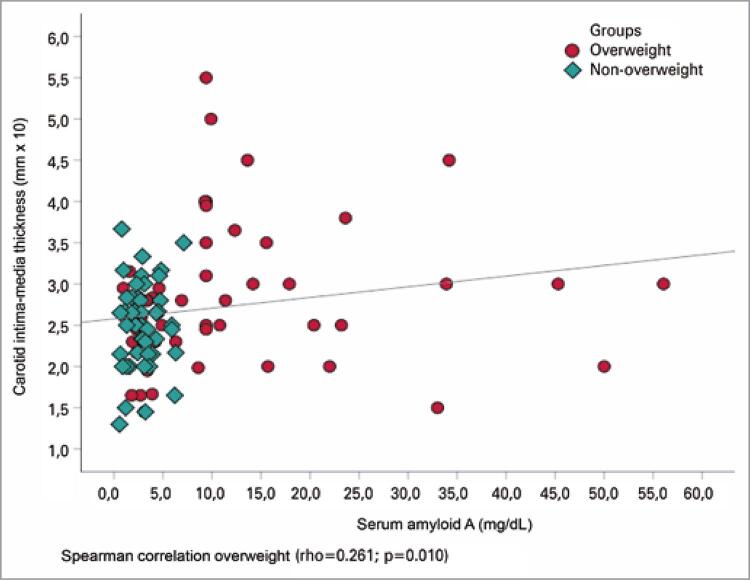
Correlation of serum amyloid A concentrations with carotid intima-media thickness

When the variables were compared between the groups continuously, the overweight group seemed to have lower values of HDL-C (p=0.004) and apolipoprotein A1 (p=0.003) and higher values of triglycerides (p=0.008), apolipoprotein B (p=0.005), total cholesterol/HDL-C (p=0.007), triglycerides/HDL-C (p=0.005), insulin (p=0.001), glycemia (p=0.028), HOMA-IR (p=0.025), us-CRP (p=0.001), SAA (p=0.001), and CIMT (p=0.029) as compared to the eutrophic group (Table 2). There was no statistically significant difference in the carotid CIMT thickness between the sexes (p=0.857) and a weak, direct, and significant correlation between SAA concentrations and the mean of the CIMT measurements (rho=0.261; p=0.010) (Figure 1).

**Table 2:** Comparison of laboratory variables and carotid intima-media thickness between overweight and non-overweight groups

Variables	Overweight	Non-overweight	p value*
Age (years)	10.8±3.2	10.8±3.1	0.924^†^
Body mass index, Z-score	2.7±1.0	-0.05±0.65	<0.001^†^
Waist-to-height ratio, cm	0.56±0.06	0.45±0.04	<0.001^†^
Total cholesterol	154.4±27.3	153.5±25.6	0.807^†^
LDL-C, mg/dL	93.1±26.8	90.2±22.8	0.562^†^
HDL-C, mg/dL	43.4±8.6	49.0±10.3	0.004^†^
Non-HDL-C, mg/dL	111.0±27.4	104.6±24.7	0.218^†^
Triglycerides, mg/dL	79.1 (62.7-107.4)	63.3 (49.9-87.9)	0.008^‡^
Apolipoprotein-B, mg/dL	85.9±22.2	73.7±19.8	0.005^†^
Apolipoprotein-A1, mg/dL	126.9±20.7	139.9±21.5	0.003^†^
Total cholesterol / HDL, mg/dL	3.68±0.88	3.23±0.72	0.007^†^
Triglycerides / HDL	1.80 (1.37-2.71)	1.36 (0.98-2.07)	0.005^‡^
LDL / Apolipoprotein -B, mg/dL	1.10±0.20	1.19±0.14	0.010^†^
Apolipoprotein-B / A1, mg/dL	1.58±0.50	1.93±0.46	0.001^†^
Insulin, uU/mL	11.7 (7.3-14.7)	5.3 (3.1-9.5)	0.001^‡^
Glucose, mg/dL	81.6±7.7	78.0±8.2	0.028^†^
HOMA-IR	2.26 (1.51-2.87)	1.01 (0.57-2.00)	0.025^‡^
us-CRP, mg/L	1.0 (0.6-2.4)	0.3 (0.2-0.9)	0.001^‡^
Serum amyloid A, mg/L	9.4 (3.4-15.5)	2.8 (1.7-3.7)	0.001^‡^
CIMT, mm	0.284±0.087	0.252±0.050	0.029^†^

^†^ Significance level of the *t*-Student test; ^‡^ Significance level of Mann–Whitney test.

HOMA-IR: homeostasis model assessment-insulin resistance; Us-CRP: ultra-sensitive C-reactive protein; CIMT: carotid intima-media thickness.

**Figure 2:**
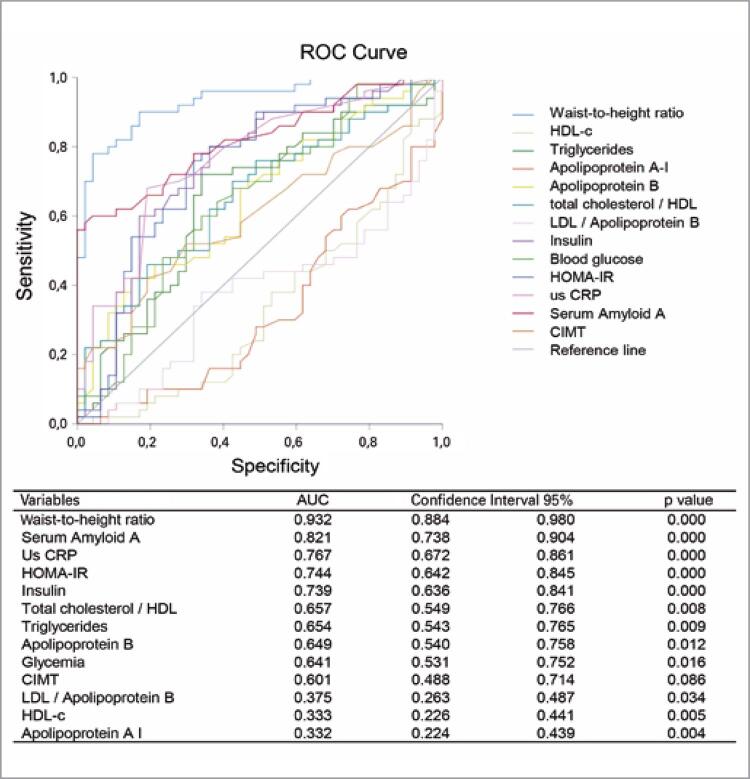
Discriminatory analysis using the ROC curve and evaluated biochemical variables with respect to the nutritional status (overweight and non-overweight) Us-CRP: ultra-sensitive C-reactive protein; HOMA-IR: homeostasis model assessment-insulin resistance; CIMT: carotid intima-media thickness.

In the combined analysis of the anthropometry, lipid and glucose profiles, inflammation markers, and the mean of the CIMT measurements, the WHtR, SAA, us-CRP, HOMA-IR, and insulin resistance had the highest discriminatory power between the overweight and eutrophic groups (AUC>0.7) (Figure 2).

**Figure 3:**
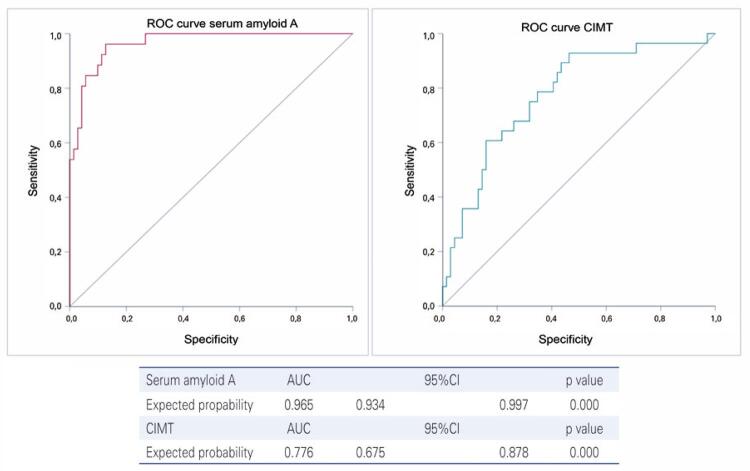
Discriminatory analysis using multivariate ROC curve with variables associated with serum amyloid A concentrations (> fourth quartile, >9.4mg/dL) and carotid intima-media thickness (> fourth quartile, >0.3mm) CIMT: carotid intima-media thickness.

The multi-ROC curves for the variables regarding lipid profile, insulin resistance, and anthropometry (overweight) were 0.965 (95%CI: 0.934-0.997) and 0.776 (95%CI: 0.675-0.878) for SAA (>9.4mg/dL) and for CIMT measurement (>0.3mm), respectively (Figure 3).

In the multivariate analysis, age (OR=1.73; 95%CI: 1.16-2.60, p=0.007), BMIz (OR=3.76; 95%CI: 1.64-8.59, p=0.002), apolipoprotein B (OR=1.10; 95%CI: 1.01-1.20, p=0.030), and CIMT (OR=5.00; 95%CI: 1.38-18.04, p=0.014) were found to be independently associated with SAA values (>9.4mg/dL) (Table 3).

**Table 3:** Logistic regression of variables associated with serum amyloid A levels (Model I) and carotid intima-media thickness (Model II)

	OR	95%CI		p value
Model I				
Age	1.739	1.163	2.602	0.007
Body mass index Z-score	3.759	1.643	8.598	0.002
Apolipoprotein-A I	0.971	0.920	1.024	0.274
Apolipoprotein-B	1.102	1.010	1.203	0.030
HOMA-IR	1.239	0.785	1.956	0.357
us-CRP	1.521	0.912	2.537	0.108
CIMT	5.004	1.387	18.048	0.014
Dependent variable: serum amyloid A (>fourth quartile >9.4mg/dL)				
Model II				
Age	0.186	0.984	1.474	0.072
Body mass index Z-score	0.253	0.906	1.832	0.159
Apolipoprotein-A1	-0.001	0.974	1.025	0.930
Apolipoprotein-B	-0.027	0.942	1.005	0.099
HOMA-IR	0.062	0.843	1.344	0.601
us-CRP	0.028	0.847	1.250	0.774
Serum amyloid A	0.052	0.988	1.123	0.114
Dependent variable: carotid intima-media thickness (>fourth quartile >0.3mm)				

HOMA-IR: homeostasis model assessment-insulin resistance; Us-CRP: ultra-sensitive C-reactive protein; CIMT: carotid intima-media thickness.

## DISCUSSION

This study showed that overweight children and adolescents (mean age = 10.8±3.16 years old) showed higher SAA concentrations than eutrophic children and adolescents. There was an independent and direct association between SAA concentrations (>9.4mg/dL) and age, BMIz, apolipoprotein B, and CIMT, suggesting a risk for cardiovascular disease.

Serum amyloid A is an acute-phase protein that increases the levels of prothrombotic and pro-inflammatory molecules. In the acute-phase response, SAA is synthesized by the liver and is transported mainly in association with HDL-c. Moreover, SAA can be found in association with lipoproteins containing apolipoprotein B. This protein is detected in adipose tissues and atherosclerotic lesions, where it can be part of the physiopathogenesis of these lesions.^(9)^ Studies have demonstrated that a high SAA concentration is associated with an increased risk of CVD.^(10)^ However, there have been no studies on pediatric age.

Children and adolescents in the overweight group presented higher concentrations of us-CRP as compared to those in the non-overweight, which is consistent with the study by Mărginean et al. in patients of a similar mean age.^(11)^ A systematic review and meta-analysis found significantly higher us-CRP values in obese individuals of both sexes and all age groups.^(12)^ Obesity is considered a low-grade chronic systemic inflammatory disease in which adipose tissue undergoes changes, increasing the secretion of pro-inflammatory adipokines and cytokines, acute-phase proteins, and alterations in macrophage functions.^(13)^

The lower values of HDL-c and higher values of triglycerides, total cholesterol, and LDL-c observed in the overweight group are consistent with another study conducted in Brazil (n=1.243, median of 12 years).^(14)^ The triglycerides/HDL ratio was higher in the overweight group. This ratio has been reported to be an early and sensitive predictor of insulin resistance and may be altered before HOMA-IR.^(15)^

Fasting glycemia, insulin, and HOMA-IR were higher in overweight and obese children and adolescents, which is consistent with other studies.^(16)^

Apolipoprotein A1 levels were lower, whereas apolipoprotein B levels were higher in the overweight group as compared to the non-overweight. A meta-analysis of eight studies with mean ages of nine to 15.7 years found an average reduction of 8.13mg/dL (95%CI: -9.09- -7.17mg/dL) and an average increase of 4.94mg/dL (95%CI: 4.22-5.67mg/dL) of apolipoprotein A1 and B in overweight and obese children and adolescents.^(17)^Apolipoprotein A1 has the ability to modulate the energy expenditure of adipocytes, reduce atherosclerosis, and lead to the accumulation of hepatic lipids, as well as inflammation; this protein is considered a protective factor for CVD.^(18)^ On the other hand, apolipoprotein B is related to atherogenic particles, and its levels are directly associated with hypertension, central obesity, and insulin resistance, increasing the risk of CVD.^(19)^

Carotid intima-media thickness is a noninvasive test that can be used to evaluate atherosclerosis early on, before the effective formation of atherosclerotic plaques.^(20)^ A recently published study with Brazilian children (n=59, mean age of 8.8 years) revealed that overweight/obese children had higher CIMT (0.49±0.07mm) than eutrophic children (0.41±0.05mm) of the same age group and both sexes.^(21)^ In agreement with our findings, most studies reported a significant increase in CIMT in obese children and adolescents compared with adequate weight controls.^(22-24)^

To our knowledge, this is the first study to show a significant and independent association between SAA and BMIz, CIMT scores, and apolipoprotein B, drawing attention to the importance of this inflammation biomarker in identifying the risk of atherosclerosis. In adult patients with type 2 diabetes, an independent association was observed between CIMT, obesity, and SAA.^(25)^

## CONCLUSION

Overweight children and adolescents have higher serum amyloid A concentrations than eutrophic children. An independent association between higher concentrations of serum amyloid A and Z-score, body mass index, apolipoprotein B, and carotid intima-media thickness was observed, indicating the importance of this inflammatory biomarker in identifying the early risk of atherosclerosis.

## References

[1] World Health Organization (WHO) (2021). Obesity and overweight.

[2] Instituto Brasileiro de Geografia e Estatísticas (IBGE) (2020). Pesquisa nacional de saúde: 2019: atenção primária à saúde e informações antropométricas.

[3] Chait A, den Hartigh LJ (2020). Adipose tissue distribution, inflammation and its metabolic consequences, including diabetes and cardiovascular disease. Front Cardiovasc Med.

[4] Wang Z, Nakayama T (2010). Inflammation, a link between obesity and cardiovascular disease. Mediators Inflamm.

[5] Yang RZ, Lee MJ, Hu H, Pollin TI, Ryan AS, Nicklas BJ (2006). Acute-phase serum amyloid A: an inflammatory adipokine and potential link between obesity and its metabolic complications. PLoS Med.

[6] Zhao Y, He X, Shi X, Huang C, Liu J, Zhou S (2010). Association between serum amyloid A and obesity: a meta-analysis and systematic review. Inflamm Res.

[7] Expert Panel on Integrated Guidelines for Cardiovascular Health and Risk Reduction in Children and Adolescents, National Heart, Lung, and Blood Institute (2011). Expert panel on integrated guidelines for cardiovascular health and risk reduction in children and adolescents: summary report. Pediatrics.

[8] Dalla Pozza R, Ehringer-Schetitska D, Fritsch P, Jokinen E, Petropoulos A, Oberhoffer R, Association for European Paediatric Cardiology Working Group Cardiovascular Prevention (2015). Intima media thickness measurement in children: a statement from the Association for European Paediatric Cardiology (AEPC) Working Group on Cardiovascular Prevention endorsed by the Association for European Paediatric Cardiology. Atherosclerosis.

[9] King VL, Thompson J, Tannock LR (2011). Serum amyloid A in atherosclerosis. Curr Opin Lipidol.

[10] Johnson BD, Kip KE, Marroquin OC, Ridker PM, Kelsey SF, Shaw LJ, Pepine CJ, Sharaf B, Bairey Merz CN, Sopko G, Olson MB, Reis SE, National Heart, Lung, and Blood Institute (2004). Serum amyloid A as a predictor of coronary artery disease and cardiovascular outcome in women: the National Heart, Lung, and Blood Institute-Sponsored Women’s Ischemia Syndrome Evaluation (WISE). Circulation.

[11] Mărginean CO, Meliţ LE, Ghiga DV, Mărginean MO (2019). Early Inflammatory Status Related to Pediatric Obesity. Front Pediatr.

[12] Choi J, Joseph L, Pilote L (2013). Obesity and C-reactive protein in various populations: a systematic review and meta-analysis. Obes Rev.

[13] Li C, Xu MM, Wang K, Adler AJ, Vella AT, Zhou B (2018). Macrophage polarization and meta-inflammation. Transl Res.

[14] Reuter CP, Silva PT, Renner JD, Mello ED, Valim AR, Pasa L (2016). Dyslipidemia is Associated with Unfit and Overweight-Obese Children and Adolescents. Arq Bras Cardiol.

[15] Behiry EG, El Nady NM, AbdEl Haie OM, Mattar MK, Magdy A (2019). Evaluation of TG-HDL Ratio Instead of HOMA Ratio as Insulin Resistance Marker in Overweight and Children with Obesity. Endocr Metab Immune Disord Drug Targets.

[16] Saeed W, Al-Habori M, Saif-Ali R, Al-Eryani E (2020). Metabolic Syndrome and Prediabetes Among Yemeni School-Aged Children. Diabetes Metab Syndr Obes.

[17] Jesus GD, Costa PR, Oliveira LP, Queiroz VA, Cunha CM, Pereira EM (2020). Body adiposity and apolipoproteins in children and adolescents: a meta-analysis of prospective studies. Arq Bras Cardiol.

[18] Su X, Peng D (2020). The exchangeable apolipoproteins in lipid metabolism and obesity. Clin Chim Acta.

[19] Han SJ, Fujimoto WY, Kahn SE, Leonetti DL, Boyko EJ (2020). Apolipoprotein B levels predict future development of hypertension independent of visceral adiposity and insulin sensitivity. Endocrinol Metab.

[20] El Jalbout R, Cloutier G, Cardinal MR, Henderson M, Lapierre C, Soulez G (2018). Carotid artery intima-media thickness measurement in children with normal and increased body mass index: a comparison of three techniques. Pediatr Radiol.

[21] Garcia J, Benedeti AC, Caixe SH, Mauad F, Nogueira-de-Almeida CA (2019). Ultrasonographic evaluation of the common carotid intima-media complex in healthy and overweight/obese children. J Vasc Bras.

[22] Lamotte C, Iliescu C, Libersa C, Gottrand F (2011). Increased intima-media thickness of the carotid artery in childhood: a systematic review of observational studies. Eur J Pediatr.

[23] Al-Drawny Z, Saleh S, El-Sammak A, Attia H (2020). Carotid Intima Media Thickness in Obese Egyptian Children and Adolescent. Egyptian J Hospital Med.

[24] Sajja V, Jeevarathnam D, James S, Rathinasamy J (2019). A study on carotid artery intima-media thickness and metabolic risk factors in overweight and obese Indian children. Diabetol Int.

[25] Liu Y, Li S, Benfu SM (2019). Association between serum amyloid A and carotid intima-media thickness in patients with type 2 diabetes. J Chinese Physician.

